# Suture granuloma extending intra-abdominally, detected five months postappendectomy

**DOI:** 10.1259/bjrcr.20200191

**Published:** 2021-05-26

**Authors:** Sondos Eladawi, Ahmed Ragab, Mohamed Kayed, Bassma Elsabaa, Marwa Khaled

**Affiliations:** 1 Radiology department, Faculty of medicine, Alexandria University, Alexandria, Egypt; 2 General surgery department, Faculty of medicine, Alexandria University, Alexandria, Egypt; 3 Pathology department, Faculty of medicine, Alexandria University, Alexandria, Egypt

## Abstract

**Objective:**

Suture granulomas are localised inflammatory reactions that develop at the site of retained suture material. They are a rare surgical complication that is sometimes radiologically challenging to diagnose, especially if the intra-abdominal is communicating with the anterior abdominal wall.

**Methods:**

The case reported here was a 22-year-old female who presented with right iliac fossa pain 5 months post-appendectomy, which turned out to be due to a suture granuloma. Ultrasonography and CT with and without contrast misdiagnosed the lesion as an abscess or less likely as neoplasm. Conclusive diagnosis was based upon histopathological examination of tissue obtained by biopsy.

**Conclusion:**

When reviewing the images of patients who present with post-operative surgical complications, it is crucial to consider suture granuloma as a distinct possibility. A definitive diagnosis saves the patient from undergoing unnecessary extensive surgeries and improves the patient experience.

## Introduction

Acute appendicitis is an acute inflammation of the vermiform appendix. Definitive management is usually by appendectomy. Different surgical techniques and incisions have been developed to improve the outcome and minimize post-operative complications. A post-operative abdominal wall mass in the region of McBurney’s incision is one of the recognised complications that follows an open appendectomy. Different post-operative abdominal wall mass conditions include post-operative suture granulomas, haematomas, keloids, hernias, abscesses and abdominal wall tumours.^1^

Suture granulomas are localised inflammatory reactions that develop at the site of retained suture material. They develop due to suture antigenicity and/or the presence of bacterial infection. Braided silk and Dacron are the most reactive suture materials; however, any suture material can cause a reaction.^2^ Suture granulomas can be superficial, with early onset, or deep, with later onset. They might develop months or even years after an intervention and can develop anywhere in the body. They can be asymptomatic, but sometimes become palpable and tender, mimicking a tumour, an abscess or a perforation. Suture granulomas have been reported in the conjunctiva post pulley fixation suture, in the surgical bed in the anterior compartment of the neck post thyroidectomy, in the left lower lung lobe post left basal segmentectomy, in the abdominal wall with intra-abdominal extension post appendectomy, on the antimesenteric side of the intestine post small bowel resection and in the scrotum post varicocelectomy. Nevertheless, suture granulomas generally occur very rarely as surgical complications.

Imaging plays a major role in the diagnosis of suture granulomas, and ultrasonography has been used as a first-line investigation technique. Using ultrasonography, suture granulomas exhibit a characteristic feature of a hypoechogenic^3^ collection with a hyperechogenic structure impeded in it with a rail-like morphology. Sometimes, some vascularity can be identified using colour Doppler. Despite the unique presentation on ultrasonography images, diagnosis is often missed and finally determined by incisional/excisional biopsy and histological correlation.^2^ Other imaging modalities can be used for a definitive diagnosis, such as contrast-enhanced CT or positron emissiontomography scans.

This report presents a case of a patient with a suture granuloma with intra-abdominal extension that showed symptoms 5 months after appendectomy.

## Clinical presentation

A 22-year-old Middle Eastern female patient presented to the local hospital with a 1-week history of moderate constant right iliac fossa pain, 5 months post-open appendectomy. Clinically, the patient described having mild continuous right iliac fossa pain that showed some tenderness to touch on clinical examination. She had no fever, no nausea or vomiting and no weight loss. Clinical examination showed no rebound tenderness. Regarding her labs, inflammatory markers, including white cell count and C-reactive protein, were at the higher end of the normal range.

## Investigations and differential diagnosis

Radiologically, ultrasonography was performed, followed by non-contrast CT abdomen scanning and then contrast CT enterocolonography. Ultrasound (5–13 MHz linear transducers) showed a hypoechoic lesion with hyperechoic foci, averaging 4 × 5.4 cm in maximum axial dimensions. The lesion extended into and was continuous with the related subcutaneous layer of the skin, and on colour Doppler, no vascularity was detected. A differential diagnosis of either an inflamed appendectomy stump or an intra-abdominal abscess was made. However, these findings were not consistent with the presenting complaint, so the decision was then made to proceed with a non-contrast CT abdomen scan.

**Figure 1.**
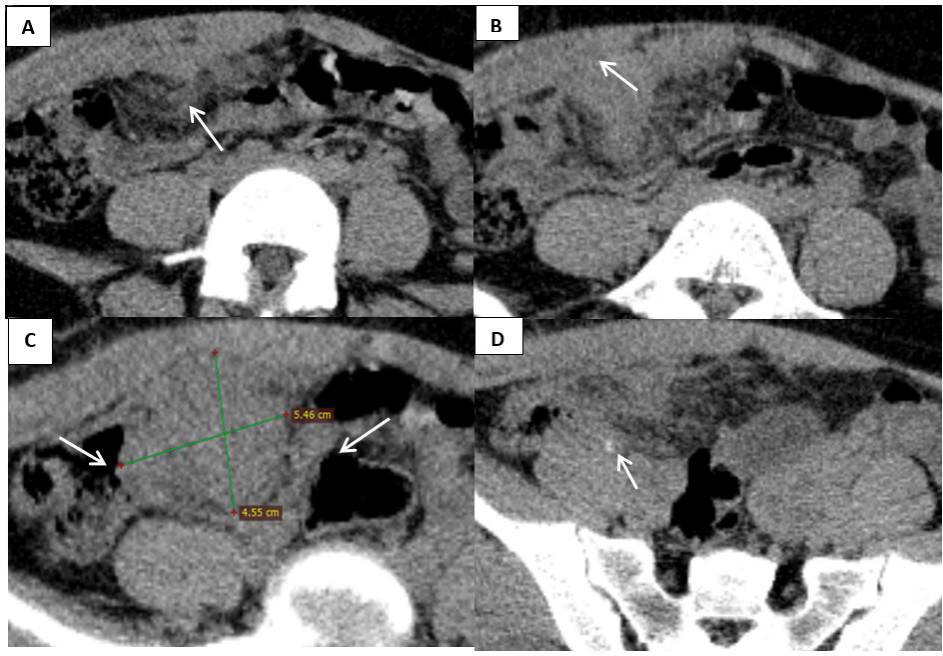
Non-contrast enhanced CT abdomen, showing a tumefactive soft tissue within the right iliac fossa measuring 4.5 × 5.5 cm in maximum axial dimensions, seen inseparable from the anterior abdominal wall (arrow, **B**), insinuated between the bowel loops (arrow, **C**), showing hyperdense focus (arrow, D), with related fat stranding (arrow, **A**).

The CT Figure 1showed an isodense mass with hyperdense foci in the right iliac fossa measuring 4.3 × 5.5 cm in maximum axial dimensions with moderate related fat stranding. The mass abutted the caecum, displacing the related ileal bowel loops laterally, and was inseparable from the related anterior abdominal wall.

These findings were not conclusive; thus, further imaging was needed. Given the close relation of the mass to the caecum and the history of appendectomy, the decision was made to proceed with CT enterocolonography rather than CT abdomen with contrast. CT enterocolonography provided better visualisation and assessment of the caecum and its relation to the mass. It was also useful to exclude the possibility of stump appendicitis. The scan was performed 2 days later. Axial cuts were taken following oral administration of neutral contrast, a water enema and intravenous contrast injection. The right iliac fossa showed a heterogeneous tumefaction with cystic component measuring 57 × 47 x 57 mm at its maximum dimensions, and enhancing wall with multiple enhancing septations as well as calcific foci within the lesion. The lesion extended to the right lower anterior abdominal wall. This was associated with related fat stranding and oedematous changes of the cecum. Abdominal organs, including the liver, hepatic biliary radicles, pancreas, spleen, kidneys and suprarenal glands were normal and dense, with no focal lesions identified. The impression reported was an intraperitoneal loculated collection with abscess formation and typhlitis rather than a neoplastic process.

## Treatment

As per the multidisciplinary team’s advice, the patient underwent surgical excision of the mass. A sample was sent to the histopathology team for pathological correlation. Further planning for the management of the patient was performed.

## Outcome and follow-up

The excised sample was examined by an experienced pathologist who reported that sections through the lesion demonstrated well-formed non-caseating granulomas made up of plump epithelioid histiocytes. These structures were surrounded by variable amounts of lymphocytes and plasma cells and were associated with robust surrounding fibroplasia. In the centre of the reaction, suture material was identified as particulate refractile blackish matter; this finding was in line with inflammatory suture granuloma. No neoplastic lesions or abscess formation were identified. An established diagnosis of suture granuloma was then made, and the patient was reassured.

The patient recovered very well after the surgical excision of the granuloma. Her pain had improved, and no further mass was identified during the 6 month follow-up appointment.

## Discussion

Suture granulomas are groups of cells that cluster at or near the site of surgical sutures as a part of the body’s response to foreign material.^2,4^ These granulomas are mostly associated with non-absorbable sutures.^5^ Suture granulomas have been reported at various times following surgery. Their duration ranges from a few days to many months or even years following surgery.^1^ The histopathological nature of the granuloma depends on the material of the suture entrapped and the reaction of the body towards it.^4^ Clinically, suture granulomas do not have typical presentations; they can be asymptomatic and are sometimes found incidentally, and symptoms depend on granuloma site, extent and size. In one case, suture granulomas appeared as a recurrent tumour on follow-up CT scans post colorectal tumour resection.^6^ External factors, such as exposure to radiation, have been reported to increase the likelihood of suture granuloma.^7^

The present case represents a rare complication of a suture granuloma with intra abdominal extension from the abdominal wall that occurred 5 months post appendectomy. A search of literature published within the last 20 years revealed only 3 similar reported cases: Matsuda et al^8^ 11 years post-operatively, Ichimiya et al^9^ 25 years post-operatively and Augustin et al^1^ 12 years after appendectomy.

With the development of different surgical incisions and appendectomy techniques, some post-operative complications have arisen, including post-operative abdominal wall mass in the region of McBurney’s incision. These complications may occur early or late after surgery. Late complications are considered rare and can include adhesive intestinal obstruction, incisional hernia and suture granuloma. Most of these complications can present as an abdominal mass.^1^

Thus, when encountering a post-appendectomy abdominal mass, a list of differential diagnoses should be considered. The mass could be primary or related to the appendectomy or to any other pathologic condition in the area. Therefore, it is of crucial importance to do a thorough clinical examination and history taking, all while employing radiological techniques, including abdominal ultrasound and contrast-enhanced multislice CT scan, to reach the correct diagnosis.^1^

In the present case, the suture granuloma diagnosis had been missed on three imaging modalities, reported by three different radiologists with different levels of expertise ranging from senior registrar to consultant. This illustrates the pressing need to keep suture granulomas in mind as a differential diagnosis for patients presenting with post-operative complications.

## Limitations

The patient’s contrast-enhanced CT abdomen scan was performed at an imaging centre that did not share the imaging data with our local picture archiving and communication system. Therefore, we had access to the written report provided by the patient but not to the image itself.

## Learning points

1. Clinicians should include suture granulomas in the differential diagnosis when imaging studies identify intra-abdominal masses in patients with post-operative abdominal complications at the operative site.
2. Ultrasonography should be the first methodology used in the diagnosis of suture granulomas. It is the least invasive technique and minimises radiation exposure and patient anxiety.

## References

[1] Augustin G, Korolija D, Skegro M, Jakic-Razumovic J. Suture granuloma of the abdominal wall with intra-abdominal extension 12 years after open appendectomy. World J Gastroenterol 2009; 15: 4083. doi: 10.3748/wjg.15.4083 19705509 PMC2731964

[2] Singh SK, Kannan N, Talwar R, Tyagi AK, Madan R, Jaiswal P, et al. Suture granuloma: a rare differential diagnosis of residual/recurrent gastrointestinal stromal tumor of stomach. Int Cancer Conf J 2016; 5: 5–8 Jan 1 (Vol. 5, No. 1, pp. 5-8). Springer Japan. doi: 10.1007/s13691-015-0216-8 31149414 PMC6485273

[3] Rettenbacher T, Macheiner P, Hollerweger A, Gritzmann N, Weismann C, Todoroff B. Suture granulomas: sonography enables a correct preoperative diagnosis. Ultrasound Med Biol 2001; 27: 343–50. doi: 10.1016/S0301-5629(00)00364-1 11369119

[4] Takeshita N, Tohma T, Miyauchi H, Suzuki K, Nishimori T, Ohira G, et al. Suture granuloma with false-positive findings on FDG-PET/CT resected via laparoscopic surgery. Int Surg 2015; 100: 604–7. doi: 10.9738/INTSURG-D-14-00140.1 25875540 PMC4400926

[5] LoCicero 3rd J, Robbins JA, Webb WR. complications following abdominal fascial closures using various nonabsorbable sutures. Surgery, gynecology & obstetrics 1983; 157: 25–7. 6222498

[6] Kim SW, Shin HC, Kim IY, Baek MJ, Cho HD. Departments of radiology, Cheonan Hospital, Soonchunhyang University, Choongnam 330-720, Korea Korean Journal of radiology.; . Korean Journal of Radiology 2009;: 313 -–8 2009 June; 10 (3): 313-318 View PDFJun;10(3):.19412522

[7] Yoshioka Y, Nakatao H, Hamana T, Hamada A, Kanda T, Koizumi K, et al. Suture granulomas developing after the treatment of oral squamous cell carcinoma. Int J Surg Case Rep 2018; 50: 68–71. doi: 10.1016/j.ijscr.2018.07.021 30086475 PMC6085222

[8] Matsuda K, Masaki T, Toyoshima O, Ono M, Muto T. The occurrence of an abdominal wall abscess 11 years after appendectomy: report of a case. Surg Today 1999; 29: 931–4. doi: 10.1007/BF02482790 10489140

[9] Ichimiya M, Hamamoto Y, Muto M. A case of suture granuloma occurring 25 years after an appendectomy. J Dermatol 2003; 30: 634–6. doi: 10.1111/j.1346-8138.2003.tb00449.x 12928536

